# The dual-crosslinked prospective values of RAI14 for the diagnosis and chemosurveillance in triple negative breast cancer

**DOI:** 10.1080/07853890.2023.2177722

**Published:** 2023-03-07

**Authors:** Ranliang Cui, Jie Zou, Yan Zhao, Ting Zhao, Li Ren, Yueguo Li

**Affiliations:** a Department of Clinical Laboratory, Tianjin Medical University Cancer Institute and Hospital, Tianjin’s Clinical Research Center for Cancer, Key Laboratory of Cancer Prevention and Therapy, National Clinical Research Center for Cancer, Tianjin, China; b Nankai University, Tianjin, China

**Keywords:** RAI14, CA15-3, triple-negative breast cancer, early diagnosis, monitoring chemotherapy efficacy

## Abstract

**Objective:**

The exploration of non-invasive biomarkers for assessing tumor response is critical to optimize treatment decisions. In this study, we aimed at determining the potential role of RAI14 in the early diagnosis and evaluation of chemotherapy efficacy in triple-negative breast cancer (TNBC).

**Methods:**

We recruited 116 patients newly diagnosed with breast cancer, 30 patients with benign breast disease and 30 healthy controls. In addition, 57 TNBC patients were collected in serum at different time points (C0, C2 and C4) for chemotherapy monitoring. The expression of serum RAI14 and CA15-3 were quantified by Elisa and electrochemiluminescence assay, respectively. Then we compared the performances of markers with the chemotherapy efficacy assessed by imaging.

**Results:**

RAI14 is significantly overexpressed in TNBC and is linked to adverse clinicopathological features such as tumor burden, CA15-3 levels and the ER, PR, and HER2 status of the patients. ROC curve analysis showed that RAI14 improves the diagnostic performance for CA15-3(AUC_RAI14_ = 0.934 *vs.* AUC_CA15-3_ = 0.836), especially embodied in early-stage breast cancer diagnosis and patients with CA15-3 negativity. Furthermore, RAI14 behaves well in reproducing treatment response which was consistent with clinical Imaging assessment.

**Conclusions:**

Recent studies showed that RAI14 has a complementary effect to CA15-3 and a test combining the two parameters can improve the detection rate of early triple-negative breast cancer. At the same time, RAI14 plays a more important role in chemotherapy monitoring than CA15-3 as the change in its concentration is in line with the tumor volume variation. Taken together, RAI14 is a reliable novel marker in the early diagnosis and chemotherapy monitoring of triple-negative breast cancer.

## 1. Introduction

Breast cancer is one of the leading malignancies causing death in women and accounted for 30% of new cancer cases in women in 2021 with an increase of still 0.5% per year [1]. It is called triple-negative breast cancer (TNBC) because of the absence of the expression of the estrogen receptor, the progesterone receptor, and the human epidermal growth factor receptor. TNBC accounts for about 15–20% of all breast cancer cases [2,3]. Compared to other subtypes, TNBC occurs at a relatively low age while at the same time being highly aggressive. Patients are prone to metastasis and recurrence and have a poor prognosis [4–6]. The intervention of surgical and cytotoxic treatment enhances the survival rate of patients [7], data has shown that the 5-year survival rate after surgery for early-stage patients can be as high as 91% while the overall survival of patients who have metastatic TNBC is only about 18 months [8–10]. Therefore, the early diagnosis and timely treatment are significant with regard to a better prognosis and survival rate of this patient group.

Chemotherapy holds an invaluable role in controlling the progression of breast cancer. While surgery is the preferred option for most patients with early-stage TNBC, preperative neoadjuvant chemotherapy results in reduced tumor size, less difficult surgery and significantly lower postoperative recurrence rates which is correlated to disease-free survival and overall survival time [11]. Alternatively, chemotherapy provides the main treatment strategy for patients in advanced stages [12]. There is a rapid development of drug research for the treatment of TNBC during recent, however, the benefit of one particular treatment regimen varies from individuals due to the heterogeneity of tumor cells and expressed tumor markers [13]. Hence, real-time and accurate assessment of the treatment response would result in a more reliable judgement of chemotherapy efficiency and is therefore vital to guide further cancer treatment decisions. The most commonly used imaging tools to assess the dynamic changes of breast cancer are MRI and CT [14]. Even though the clinical applications are widespread, there are drawbacks that cannot be ignored, such as high costs, long scanning time, and the possibility of exposure to contrast agents, etc. Moreover, radiation is associated with a risk of inducing tumor formation [15]. Thus, MRI and CT fail to enable real-time monitoring of the tumor leading to potential delays in treatment response evaluation. In contrast, blood tests, as more convenient and non-invasive alternatives, are far more accepted by patients and have been routinely used in clinical biomarker determination.

To compensate for the lack of current efficacy monitoring tools, a large number of clinical research projects focusing on biomarker development have been conducted in the past decades. CEA and CA15-3 are the most commonly used serum markers in the diagnosis and treatment of breast cancer, while their clinical applications still remain controversial [16–19]. Persistent high expression of the two is linked to the recurrence and chemoresistance of breast cancer while detecting the concentrations is regularly applied to therapeutic efficacy monitoring [20,21]. However, studies have shown that the clinical application of CA15-3 and CEA in TNBC is limited [22]. Therefore, there is an urgent need to explore new biomarkers for effective TNBC screening as well as timely feedback of the treatment efficacy.

Previous studies [23,24] have shown that overexpression of carboxypeptidase N1 promotes metastasis of breast cancer cells, which is of great importance for clinical diagnosis and chemotherapy monitoring of breast cancer. Furthermore, we found that RAI14 and carboxypeptidase N1 are interacting proteins and that upregulation of RAI14 expression may be involved in regulating the proliferation and invasion of breast cancer cells [25,26]. RAI14 was first discovered in human retinal pigment epithelial cells as an actin-binding protein which participates in physiological processes such as the regulation of cell polarity and transport of spermatozoa [27]. Recent studies showed that RAI14 is overexpressed in gastric, esophageal, ovarian, lung cancer, and other malignant tumors with a significant role in the development of tumors [28–31]. At the same time, another research [32] have proposed that the expression of RAI14 protein in patients with hand, foot and mouth disease is correlated with the expression of DDX58, which plays an important role in the progression of hand, foot and mouth disease caused by enterovirus 71. Subsequently, several literatures [33,34] also have reported that the prognostic scoring system established by RAI14, AXL, and NOX4 genes can accurately predict the prognosis of gastric cancer patients and their sensitivity to immunotherapy; and another Risk Score model based on 5 glycolysisimmume-related genes, including RAI14, was reliable in predicting the prognosis of osteosarcoma. Therefore, RAI14 is of great exploration value in the development and prognostic assessment of disease. RAI14 expression levels are also significantly upregulated during the progression of breast cancer, especially in TNBC, where RAI14 expression levels are markedly higher as detected by database analysis. These high RAI14 levels seem to be associated with tumor immune cell infiltration and indicate adverse outcomes for the patients. However, all current studies on RAI14 were conducted in tumor tissues and cell lines. We have little knowledge of the features and clinical significance of serum RAI14 expression levels. Accordingly, it remains to be investigated whether serum RAI14 is suitable as a biomarker for clinical screening and efficacy tracking of TNBC treatment.

Thus, we included breast cancer (BC) patients, benign breast disease (BBD) patients and healthy controls in this study to evaluate the prevalence of RAI14 in breast cancer and its correlation with the clinicopathological parameters of patients to determine whether RAI14 can be considered as a serum marker for TNBC and as such be applied in the clinical diagnosis of TNBC along with CA15-3. We also evaluated the value of RAI14 in efficacy monitoring by investigating the variation pattern of serum RAI14 concentrations in TNBC patients who followed chemotherapy cycles through longitudinal efficacy assessment.

## 2 Materials and methods

### 2.1. Patients’ background

#### 2.1.1. Prevalence assessment research

The study was conducted at Tianjin Medical University Cancer Hospital from November 2021 to March 2022. Firstly, information was collected from patients’ medical records, screening patients with breast cancer and benign breast disease who were confirmed by pathological examination and did not receive any treatment, and the women who suffered from severe cardiovascular and cerebrovascular diseases, vital organ dysfunction were also excluded, also the pregnant or of lactation ones. A total of 176 participants were eventually included and recorded as 001-176, comprising 116 individuals with first diagnosed BC at different clinical stages, 30 patients with BBD and 30 age-matched healthy individuals (co-recorded with patients with BBD as controls). Subsequently, we collected preoperative or pre-chemotherapy serum specimens from patients (excluding hemolyzed and jaundiced ones) and rapidly froze them at −80 °C for use. We staged patients according to the American Joint Committee on Cancer (AJCC) TNM staging criteria and named AJCC stages I to IIIA as early stages and AJCC stages IIIB, IIIC and IV as advanced stages. After that, the clinical information including age, tumor size, clinical stage, and ER, PR, and HER2 status were collected, and we performed molecular typing of BC patients based on the Chinese Anti-Cancer Association Guidelines and Specifications for Breast Cancer Diagnosis and Treatment (2021 edition). Clinical case information for patients and controls was summarized in Supplementary Table S1.

#### 2.1.2. Chemotherapy monitoring study

To carry out the efficacy monitoring research, a total of 57 BC patients receiving chemotherapy were included and numbered 001-057, excluding those who did not receive regular chemotherapy, or had other malignancies, as well as someone with incomplete medical records based on clinical data. Later on, we collected serum samples (excluding hemolyzed and jaundiced specimens) from patients before treatment (C0, baseline), after the 2nd cycle of chemotherapy (C2) and the 4th cycle (C4), which were rapidly stored in the freezer at −80 °C. Meanwhile, to assess the efficacy of the treatment, patients underwent additional imaging (B-mode ultrasound) after receiving chemotherapy. The response to treatment of C2 and C4 was also determined following the Response Evaluation Criteria in Solid Tumors (RECIST) version 1.1, which defines partial remission (PR) as the sum of the longest diameters of all measurable target lesions ≥30% below baseline; the appearance of new lesions or a relative increase of ≥20% compared to the diameter and minimum value of the target lesions during the study along with an absolute increase of >5 mm was considered as disease progression (PD). We monitored the patients for long-term chemotherapy efficacy while keeping complete records of disease progression. The clinical information in this study was summarized in Supplementary Table S2.

### 2.2. Experimental methods

#### 2.2.1. The stability test of serum RAI14

The plasma from 5 patients was divided into 7 aliquots (100 µL each) and numbered as 1–7. No. 1 was left without any treatment; Nos. 2–4 were left at room temperature, 4 °C, and −20 °C for 24 h, separately; while Nos. 5–7 were repeatedly freeze-thawed 1, 2, and 3 times, respectively. After the above processing the RAI14 and CA15-3, CEA levels were examined in samples 1–7 under the same conditions.

#### 2.2.2. Determination of serum RAI14 concentration

The serum RAI14 concentration was measured by ELISA in patients and controls. Using a commercial kit (Shanghai Jianglai Biotechnology Co., Ltd, China), we added 100 µL of serum and standard solution to a 96-well microplate coated with capture antibody, plus biotinylated antibody and enzyme conjugate to form a complex, referring to the instructions. Finally, the TMB substrate was added and the concentration of RAI14 was calculated by detecting the absorbance at 450 nm using an enzyme-linked immunoassay instrument (Thormo, Shanghai Instruments Co., Ltd).

#### 2.2.3. Examination of CA15-3, CEA, CA125

The Roche Cobas 801 immunoassay equipped with special reagents was chosen for the determination of serum CA15-3, CEA and CA125 by electrochemiluminescence immunoassay. And it’s expressed negatively when CA15-3, CEA and CA125 in serum are below 25 U/mL, 5 µg/L and 35 U/L respectively.

#### 2.2.4. Assay of serum LDH

We used a colorimetric method to detect serum LDH concentration by a Roche Cobas c701 analyzer containing specialized reagents. Serum LDH has a normal range that is 120–250 U/L.

### 2.3. Statistical analysis

The laboratory data from this study were processed by statistical software SPSS 25.0, GraphPad Prism 9.0 and MedCalc 18.2.1 for analysis. The clinicopathological information of the patients was statistically described in the form of categorical variables and analyzed by a chi-square test. Continuous quantitative data obeying normal distribution were expressed as mean ± SD, and independent samples t-test was used for comparison between groups, while the correlation between two variables was determined *via* Pearson; the median indicated non-normally distributed quantitative data, using Mann–Whitney *U* test for comparison between groups and Spearman for correlation analysis of two variables. A ROC curve of the diagnostic efficacy of markers and the value of their rate of concentration decrease in the assessment of efficacy was chosen. *p* < .05 means that the analysis was statistically significant.

## 3. Results

### 3.1. Features of RAI14 expression in breast cancer

**Figure 1.**
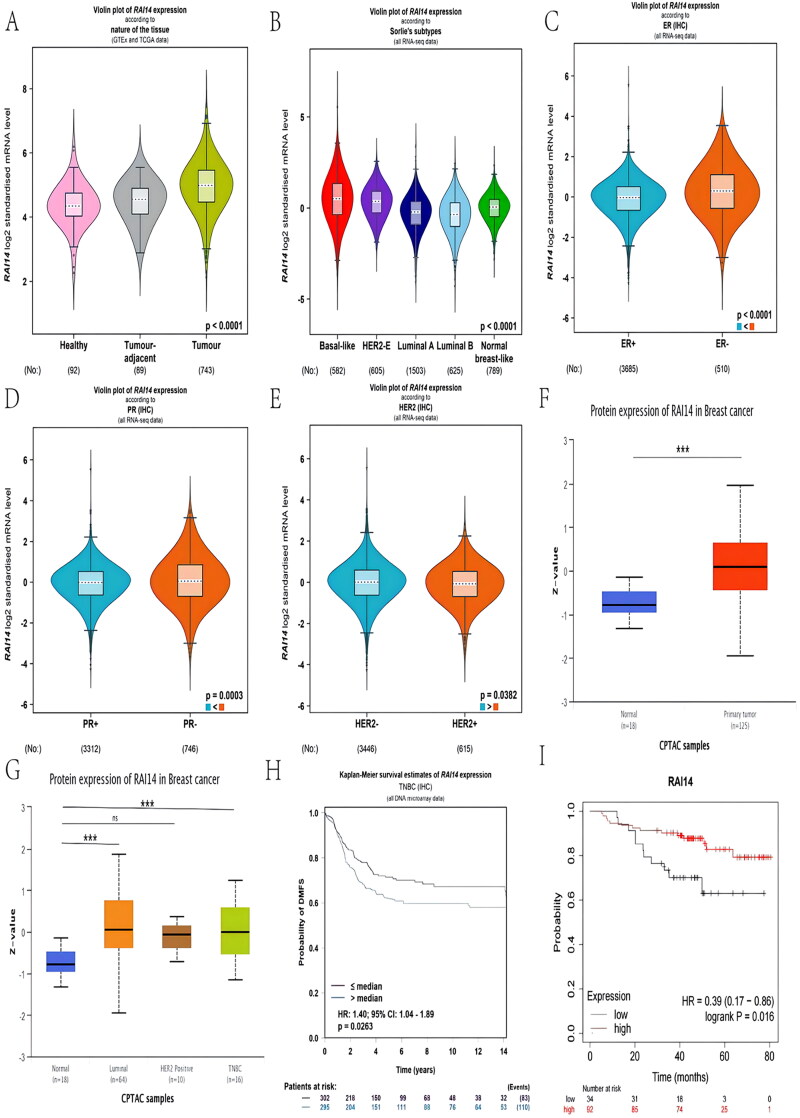
Expression characteristics of RAI14 in breast cancer. (A) Expression of RAI14 mRNA in BC tumor tissues and non-tumor tissues (BC-GenExMiner V4.8); (B) Relative expression levels of RAI14 mRNA in each BC sub-type (BC-GenExMiner V4.8); RAI14 mRNA expression features in patients with different ER (C), PR (D), ER2 (E) status (BC-GenExMiner V4.8); (F) Analysis of RAI14 protein expression in BC tumor and non-tumor tissues (UALCAN database); (G) Comparison of RAI14 protein expression in various BC isoforms (UALCAN database); Kaplan–Meier curves of DMFS (H) and OS (I) in TNBC patients according to the different levels of RAI14 based on the log-rank statistic test (BC-GenExMiner). *: *p* < .05, **: *p* < .01, ***: *p* < .001, ****: *p* < .0001, ns: no statistically significant difference.

Firstly, we found that RAI14 and CPN1 are interacting proteins (Figure S1) and explored the expression characteristics of RAI14 in breast cancer tissues by employing The Cancer Genome Atlas database. As shown in Figure 1(A), the relative RAI14 mRNA levels were significantly higher in breast cancer tissues compared to non-tumor tissues and its expression correlated with patients’ ER, PR, and HER2 status (Figure 1(C–E)). RAI14 expression was significantly upregulated when ER, PR, or HER2 expression was negative. Moreover, RAI14 mRNA was obviously expressed at a higher level in TNBC than other breast cancer subtypes (Figure 1(B)) which was in accordance with the results of proteomic analysis by CPTAC (Figure 1(F,G)). Besides, the increased expression of RAI14 correlated with the prognosis of the patients. The DMFS and OS of TNBC patients with high levels of RAI14 were noticeably shorter than those of healthy subjects (Figure 1(H,I)) so that the elevated level of RAI14 can predict a poor outcome.

### 3.2. Stability assessment of RAI14 in serum

The serum concentration of RAI14, CA15-3, and CEA was measured by placing split samples of 5 TNBC patients’ sera at different temperatures (room temperature, 4 °C, −20 °C) for 24h or after 1, 2, or 3 freeze-thaw cycles. In contrast to the varying degrees of CA15-3 and CEA (Figure S2(B,C,E,F)), we discovered that after subjecting the sera to different temperatures or to repeated freeze-thaw cycles (2–3), the level of RAI14 expression did not change more than 15% compared to the original concentration (Figure S2(A,D)).

### 3.3. Rai14 is differentially expressed in breast cancer patients and healthy controls

**Figure 2.**
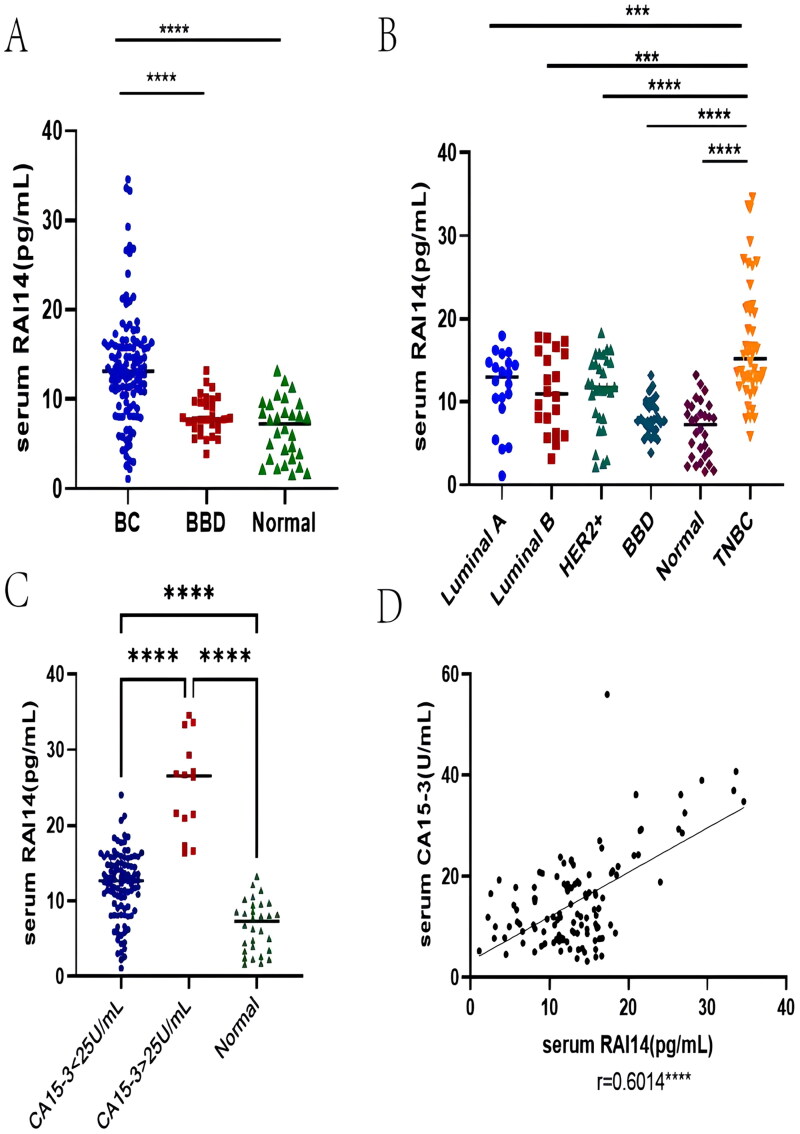
Features of serum RAI14 expression in BC. (A) The difference of RAI14 expression in BC patients, BBD patients, and healthy individuals; (B) Serum RAI14 expression in various BC subtypes compared; (C) Characterization of serum RAI14 expression in BC patients with various CA15-3 levels; (D) Correlation analysis of serum RAI14 and CA15-3. *: *p* < .05, **: *p* < .01, ***: *p* < .001, ****: *p* < .0001.

We subsequently validated the upregulation of RAI14 in breast cancer. Comparative results showed that serum RAI14 levels were statistically higher in BC patients than in healthy individuals and BBD patients (*p* < .0001, Figure 2(A)). By comparing all subtypes (Figure 2(B)), we found that TNBC patients had the highest RAI14 levels (all *p* less than .0001). Furthermore, there was a positive correlation between RAI14 and CA15-3 (*r* = 0.6014, *p* < .0001, Figure 2(D)). Based on the CA15-3 concentrations, we divided the patients into a CA15-3 negative and a CA15-3 positive group and afterwards analyzed whether there was a statistical difference in RAI14 expression between the two. As d isplayed in Figure 2(C), in both groups, RAI14 expression levels were markedly higher than those of healthy individuals (*p* < .0001) with the levels of CA15-3 positive patients being significantly higher than those in the CA15-3 negative patient group (*p* < .0001).

### 3.4. Factors affecting the RAI14 levels

**Figure 3.**
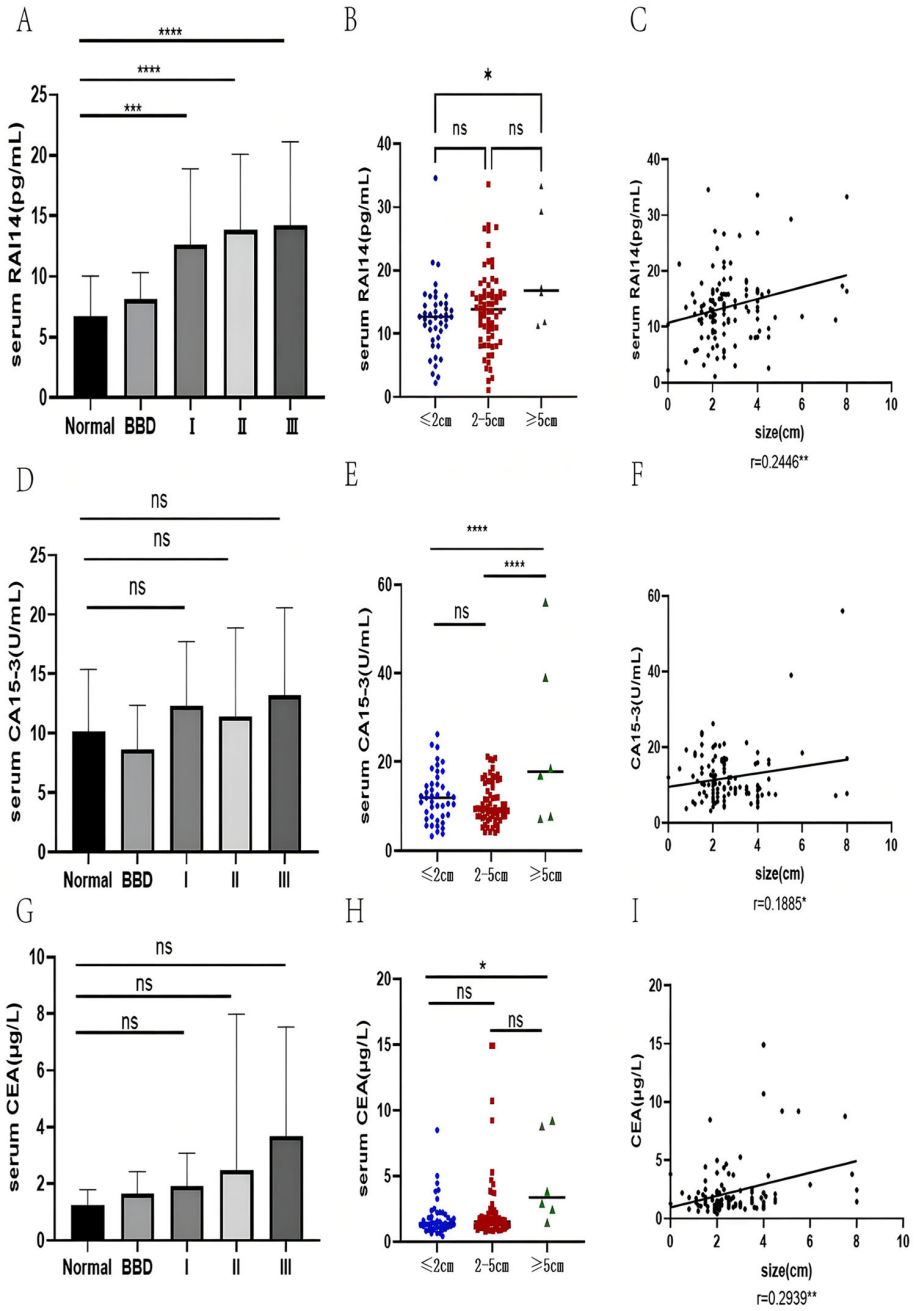
Analysis of the expression feature of RAI14, CA15-3 and CEA in BC with different clinical stages and tumor sizes. (A) RAI14 serum levels in healthy subjects, BBD patients and BC patients (at different clinical stages); (B) RAI14 concentration in BC patients with variable tumor size; (C) Correlation analysis of serum RAI14 level and tumor diameter; (D) CA15-3 serum levels in healthy subjects, BBD patients and patients of diverse clinical stages of BC; (E) Serum CA15-3 concentrations in BC patients in various tumor dimensions; (F) The correlation between serum CA15-3 level and tumor diameter; (G) CEA serum concentrations among healthy subjects, BBD patients and patients with a variety of clinical stages of BC; (H) RAI14 expression in BC patients showing a range of tumor sizes; (I) Relationship between the levels of serum CEA and tumor size. *: *p* < .05, **: *p* < .01, ***: *p* < .001, ****: *p* < .0001, ns: no statistically significant difference.

We explored the clinicopathological factors related to RAI14 expression *via* univariate analysis. The results are presented in Table 1 which shows that RAI14 levels are obviously affected by tumor size (*p* = 0.020). Patients with larger tumor diameters had higher RAI14 levels (*p* = .0081, *r* = 0.245). The expression was also correlated with the ER, PR, and HER2 status, as well as the LDH levels(all *P* less than 0.05), whereas patients’ age, clinical stage, and lymph node metastasis had no effect on the levels. In addition, we categorized the patients according to the clinical stage into three groups of stage I, II and III and found that although the levels were significantly higher in each group compared to healthy controls, no statistical difference among the stages was found which was partly different from CA15-3 and CEA (Figure 3(A,D,G)). Following the same analysis, we found that the BC tumor size-induced changes in RAI14 levels was similar to CA15-3 levels (Figure 3(B,C,E,F,H,I)) while there was a strong correlation between the two biomarkers’ levels (*r* = 0.6014, *p* < .0001, Figure 2(D)), but not among the CEA and CA125 levels.

**Table 1.** Relative factors in the prevalence assessment study.

Variables		*N*	RAI14 concentration （pg/mL）	*p* value
Age				
	<50	47	129.76 ± 55.75	.363
	≥50	69	140.76 ± 68.45	
Size (cm)				
	≤2	43	124.11 ± 55.37	.020*
	2–5	67	138.51 ± 63.14	
	≥5	6	198.98 ± 92.47	
TNM stage				
	I–IIIA	104	134.91 ± 61.93	.490
	IIIB–IV	12	148.36 ± 78.56	
Lymph node metastases				
	Absent	46	142.26 ± 61.82	.416
	Present	70	132.39 ± 64.86	
ER expression				
	Positive	40	114.38 ± 46.32	.007**
	Negative	76	147.84 ± 68.50	
PR expression				
	Positive	34	115.14 ± 46.46	.020*
	Negative	82	145.07 ± 67.78	
HER2 expression				
	Positive	50	112.81 ± 45.52	<.0001****
	Negative	66	154.10 ± 69.60	
CA15-3 expression				
	Negative	72	120.49 ± 44.78	<.0001****
	Positive	44	251.49 ± 62.70	
CEA expression				
	Negative	78	133.87 ± 62.71	.131
	Positive	38	169.13 ± 70.55	
CA125 expression				
	Negative	84	136.14 ± 64.77	.462
	Positive	32	138.50 ± 47.86	
LDH expression				
	Negative	93	135.94 ± 60.46	.018*
	Positive	23	149.81 ± 165.70	

*p<0.05,**p<0.01, ****p<0.0001.

### 3.5. The application of RAI14 in TNBC diagnosis

**Figure 4.**
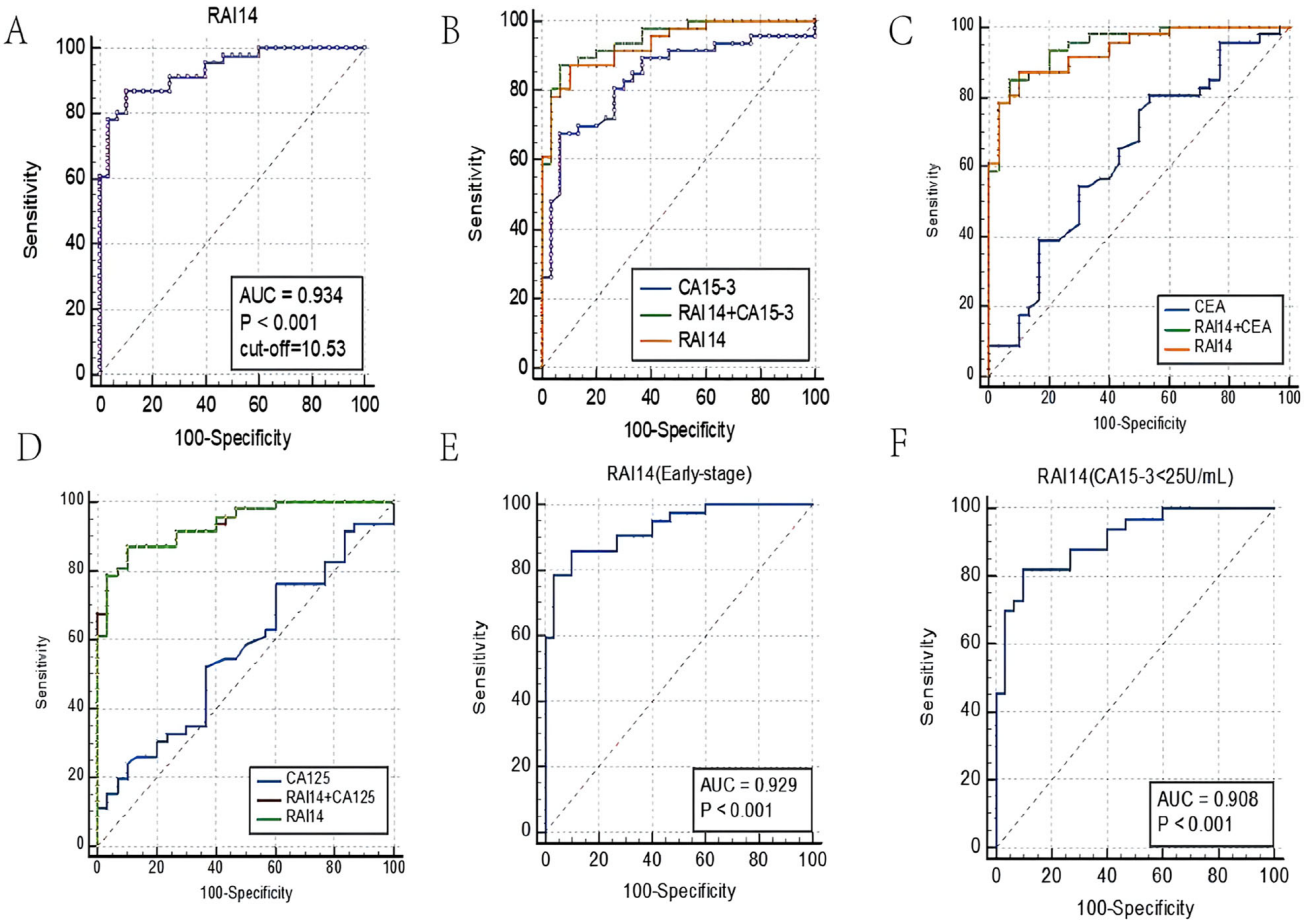
Diagnostic efficacy of serum biomarkers for TNBC. (A–D) ROC curves of serum RAI14 (A), CA15-3 (B), CEA (C) and CA125 (D) alone and in combination for the diagnosis of TNBC; (E) Diagnostic value of serum RAI14 for early TNBC; (F) Efficacy of serum RAI14 to differentiate between CA15-3 negative TNBC patients and healthy individuals.

We then plotted the RAI14 ROC curves for diagnostic purposes which are shown in Figure 4(A). The diagnostic efficacy was superior to that of CA15-3, CEA, and CA125 (AUC_RAI14_ = 0.934 vs AUC_CA15-3_ = 0.836, AUC_CEA_ = 0.636, AUC_CA125_ = 0.566, Figure 4(B–D), Table S3). The ROC curve analysis also showed that RAI14 has a good diagnostic performance for patients in an early TNBC stage (AUC_RAI14 (Early-stage)_ = 0.929, *p* < .0001, Figure 4(E). CA15-3 negative patients still highly expressed RAI14, making this parameter of high diagnostic value for TNBC patients (Figure 4(F), AUC_RAI14（CA15-3<25U/mL）_ = 0.908, *p* < .0001). Co-diagnostic ROC curve of RAI14 together with traditional markers showed that the combination of RAI14 with CA15-3, CEA, and CA125 dramatically improved the diagnostic efficacy of TNBC (Table S3).

### 3.6. Application of serum RAI14 in monitoring chemotherapy efficacy

#### 3.6.1. The dynamic changes of markers’ levles during chemotherapy

We judged the effectiveness of chemotherapy during the treatment based on a comprehensive clinical evaluation of tumor changes in the C0 and C4 stages and divided the patients into a PR and a non-PR group accordingly. After dosing, an overall significant decrease in RAI14 concentrations was observed in the PR group and the RAI14 expression characteristics were similar to those of CA15-3 and CEA in the prevalence assessment study. Therefore, we hypothesize that the change pattern of RAI14 during chemotherapy is consistent with that of CA15-3, i.e. patients with a high expression prior to treatment display decreased serum levels after effective chemotherapy; in case of ineffective treatment with an associated disease progression, accordingly, the RAI14 expression increases. To further confirm the above hypothesis, we stratified the patients of the PR group into a cPR and iPR group depending on whether RAI14 varied regularly during chemotherapy. Among them, the RAI14 levels in the cPR group showed a regular trend with chemotherapy cycles while this trend was irregular in the iPR group. As shown in Figure S3(A), there was generally a significant decrease of RAI14 in the cPR group with increasing chemotherapy cycles (*p* < .05), while no markable decrease was seen in the non-PR group (Figure S3(B)). The changing regularity of CA15-3 and CEA was similar to the above results, but no statistically significant differences were found in the CA15-3 and CEA changes in the cPR group (Figure S3(C–F)).

#### 3.6.2. Correlation analysis of RAI14 level changes and evaluation of therapy efficacy

**Figure 5.**
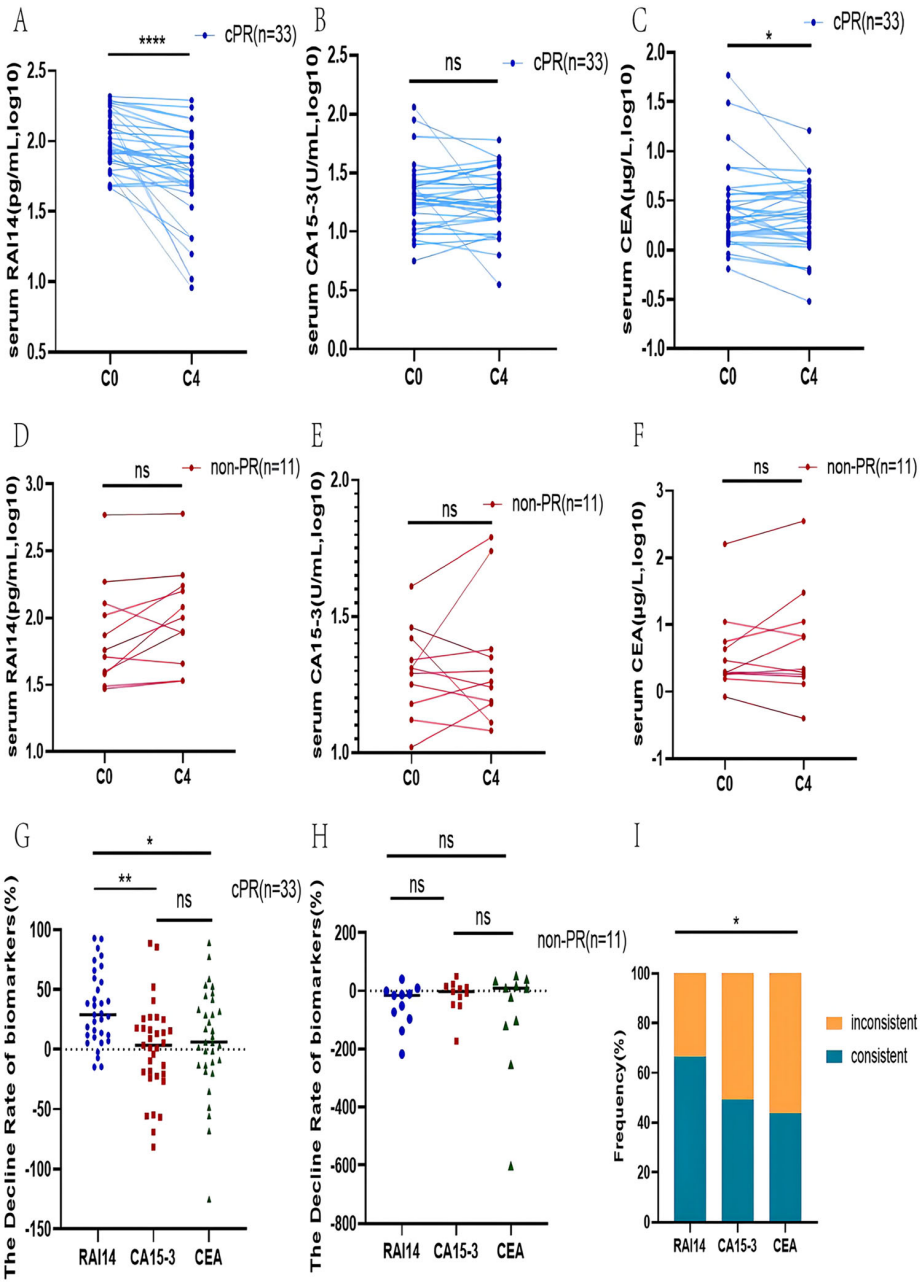
Change the pattern of serum marker levels pre-and post-chemotherapy of patients. (A–C) Trends in serum concentrations of RAI14 (A), CA15-3 (B) and CEA (C) in the cPR group in the C0–C4 phase. (D–F) Alteration pattern of RAI14 (D), CA15-3 (E) and CEA (F) in the non-PR group at C0–C4 stage. (G) Distribution of RAI14, CA15-3, and CEA decline rates in the C0–C4 phase in the cPR cohort. (H) Analysis of the distributions of RAI14, CA15-3, and CEA decline rates in patients in the iPR group at C0–C4. (I) Consistency of changes in concentration of each marker with chemotherapeutic efficacy.

We further analyzed the relationship between the changes in RAI14 levels and the clinical efficacy during the study period in both the cPR and the non-PR group and found that its changes accurately reflected the therapeutic effect. Patients treated effectively were found to display reduced RAI14 levels in stage C4 compared to C0 (*p* < .0001, Figure 5(A)) and the decline rate was higher than that of CA15-3 and CEA (Figure 5(B,C,G)) with statistically significant differences (*p* < .05), whereas no apparent decrease in serum RAI14, CA15-3, and CEA levels was seen in the non-PR group (Figure 5(D–F,H)). Subsequent analysis of whether the trend of each marker was consistent with the clinical evaluation after drug administration revealed that RAI14 levels more accurately reflected the treatment efficacy than CA15-3 and CEA levels (*p* < .05, Figure 5(I)).

#### 3.6.3. Monitoring the role of RAI14 decline rate in the efficacy assessment

**Figure 6.**
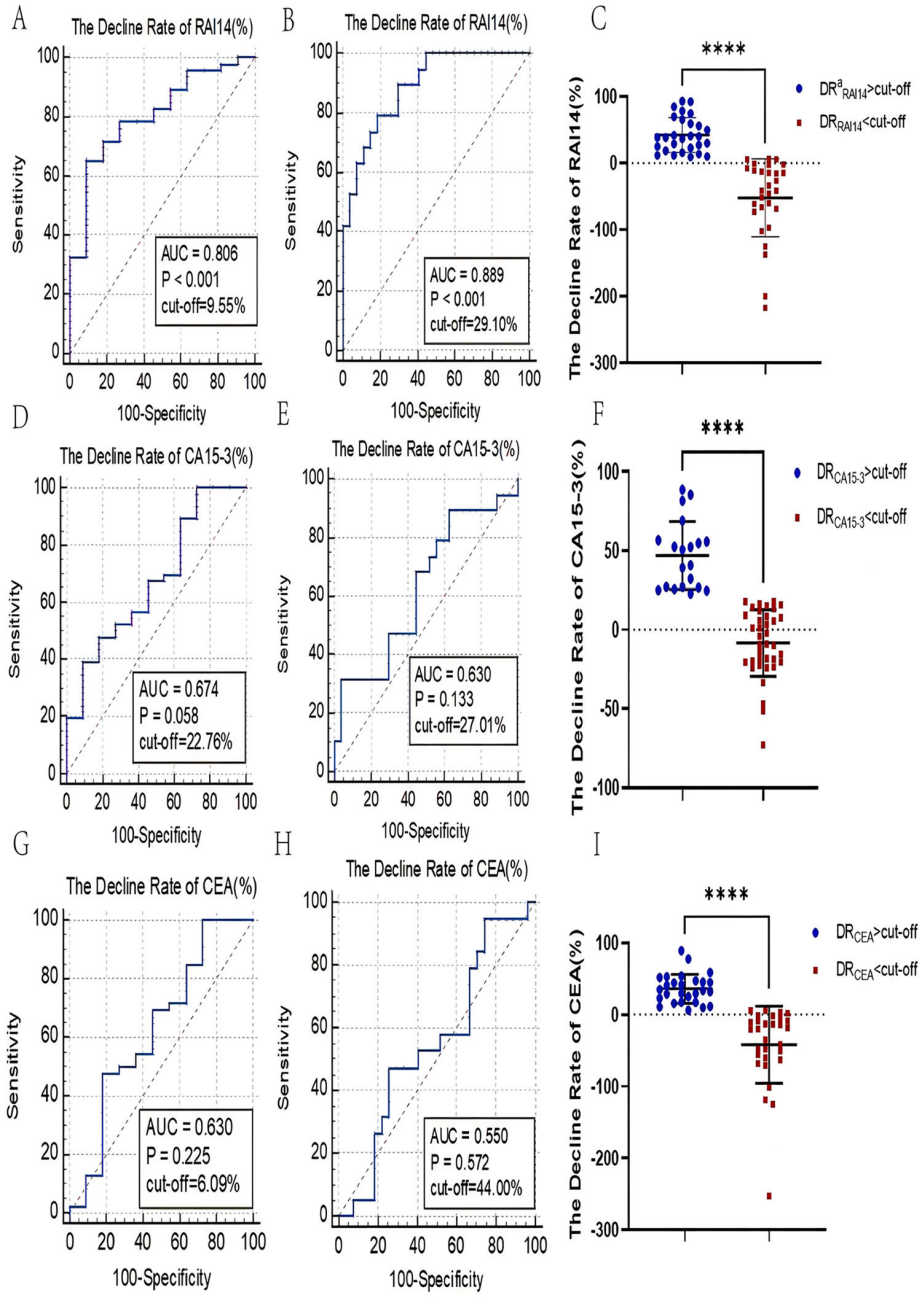
Ability to monitor chemotherapy by the rate of decline in serum marker concentrations. (A,D,G) ROC curves of RAI14 (A), CA15-3 (D), and CEA (G) decline rates distinguishing PR, non-PR patients; (B,E,H) Efficacy of RAI14 (B), CA15-3 (E), and CEA (H) decline rates to distinguish PR' and SD classes; (C,F,I) Detection of RAI14 (C), CA15-3 (F), and CEA (I) decline rates in PR, non-PR groups.

After calculating the decrease rate of each marker in both, the PR and the non-PR group, after chemotherapy, serum RAI14 levels that decreased by more than 9.55% based on the ROC curves, were considered as being effective in therapy (Figure 6(A), AUC_decline rate of RAI14_ = 0.806, *p* < .001). Then, we classified patients who did not suffer from disease progression into a PR'(partial response) and an SD(stable) group and showed that the treatment was highly effective if the RAI14 concentration dropped by more than 29.10% (Figure 6(B), AUC_decline rate of RAI14_ = 0.889, *p* < .001). In contrast, the variation of the CA15-3 and CEA levels have less ability to differentiate patients with regard to treatment efficacy (AUC_decline rate of RAI14_ = 0.806 vs AUC _decline rate of CA15-3_ = 0.674, AUC_decline rate of CEA_ = 0.630; AUC_decline rate of RAI14_ = 0.889 vs AUC_decline rate of CA15-3_ = 0.630, AUC_decline rate of CEA_ = 0.550, Figure 6(D,E,G,H)). The general distribution of the decline rates of RAI14, CA15-3, and CEA after chemotherapy is demonstrated in Figure 6(C,F,I), respectively.

#### 3.6.4. Validation of the assessment ability to derive the treatment efficacy from the RAI14 decline rate

We then listed four patients and verified the accuracy of their RAI14 concentration decline rate for efficacy assessment. Two patients experienced disease progression at C2 and C4, respectively, as shown in Figure S4(A,B), with correspondingly elevated RAI14 levels. In parallel, the RAI14 serum levels of both decreased at the C2 and C4 stages (the DR of RAI14_C2_ = 55.10%, the DR of RAI14_C4_ = 14.53%), and the therapy was determined to be significantly effective and effective, respectively, which is consistent with the clinical assessment results. The other two patients had a continuous decreasing trend of RAI14 levels during chemotherapy (the reduction rate was higher than 29.10%) with both treatments having been classified as significantly effective and that resembled the clinical assessment (Figure S4(C,D)).

### 3.7. Comparison of RAI14 with imaging in several cases

**Figure 7.**
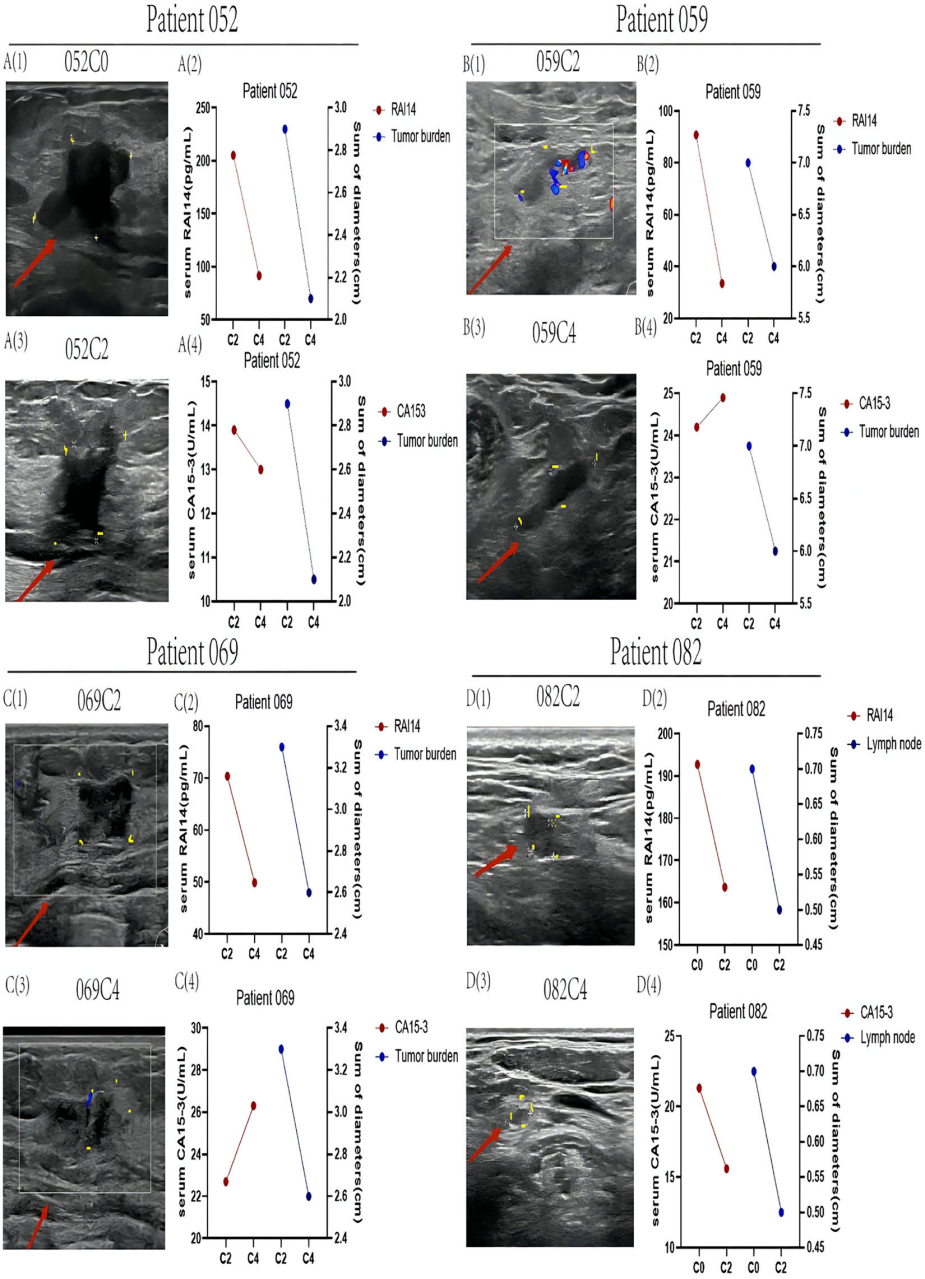
Variations of RAI14, CA15-3 and tumor size over chemotherapy treatment periods. A(1), A(3), B(1), B(3), C(1), C(3), D(1), D(3): Variation of tumor volume after 2 cycles of chemotherapy; A(2), B(2), C(2), D(2): Patients’ RAI14 level changes after 2 cycles of chemotherapy; A(4), B(4), C(4), D(4): Variation of CA15-3 concentration in patients after 2 cycles of chemotherapy.

The changes in tumor size, RAI14, and CA15-3 levels in parallel to the chemotherapy cycles in the other four patients were analyzed in a comprehensive manner. The results are displayed in Figure 7. Comparing them to each other, the highest degree of a RAI14 decline was seen in patient 059 (the DR of RAI14_059_ = 63.01% vs the DR of RAI14_052_ = 55.00%, the DR of RAI14_069_ = 29.13%, the DR of RAI14_082_ = 15.08%, Figure 7(A2, B2, C2, D2). Simultaneously, there was a noticeable decrease in the serum RAI14 level and tumor size after effective chemotherapy of all patients listed (Figure 7(A1–3, B1–3, C1–3)). In contrast, CA15-3 varied relatively randomly (Figure 7(A4–D4)), including two patients whose serum CA15-3 levels even tended to increase after chemotherapy (Figure 7(B4, C4)), which was disparate to the imaging findings.

### 3.8. Relationship between the baselines of RAI14 and the therapeutic efficacy

From the ROC curves, we could show that patients with RAI14_C0_ levels that were higher than the cut-off (cut-off = 57.45 pg/mL), could be diagnosed as having a better treatment efficacy (AUC = 0.706, *p* < .05, Figure S5(A)). Next, the patients were divided into 2 groups on the basis of comparing the baseline and the cut-off RAI14 values. The RAI14 changes after medication and the distribution of the decrease rate in each group are presented in Figure S5(B,C), which indicate that patients with high baselines had better results after treatment (*p* < .05). Correlation analysis of RAI14 baselines and decline rates revealed a positive result (Figure S5(D)), i.e. the higher the patients’ concentrations at C0, the greater their decline after receiving chemotherapy (*r* = 0.3036, *p* < .05), showing that the patients’ initial RAI14 levels were relevant to later therapy outcomes.

### 3.9. Reasons for unintended changes in RAI14 levels

The changing pattern of marker concentrations in the iPR group is given in Figure S3(A). Patients in this group exhibited random fluctuations in RAI14 levels during treatment, which were stratified into three groups, as seen in Figure S6(A–C). Firstly, patients in the chemoresistant group tended to have an increased RAI14 level in the C0–C2 phase, followed by a decrease in some patients (Figure S6(A)). Figure S6(B) demonstrates how RAI14 levels changed with the chemotherapy cycle in the myelosuppressed group. We found that there was an overall decreasing trend in the RAI14 levels in the C0–C2 phase of these patients. Finally, among patients with abnormal liver function, a marked increase of serum RAI14 levels was observed throughout chemotherapy (Figure S6(C)).

## 4. Discussion

TNBC is a subtype of breast cancer that is negative for ER, PR, and HER2 status. It is highly aggressive and prone to metastasis and recurrence with a worse prognosis than any other breast cancer subtype [35,36]. Imaging is the main tool for TNBC screening at present but due to its low sensitivity and specificity, microscopic lesions are easily missed. Although being the gold standard, pathological diagnosis also has some disadvantages such as being invasive, delivering only a limited sample amount and being time-consuming, therefore, more accurate and efficient screening methods need to be explored [37–39].

Clinical treatment of TNBC is still dominated by chemotherapy but owing to tumor heterogeneity, there are differences in patients’ response to the same drug regimen [13]. Thus, real-time monitoring of treatment efficacy is essential for the development and adjustment of medication protocols. Imaging methods, such as MRI and CT, are the most commonly applied ones for tumor monitoring. However, they are not only costly but also harbor an inherent radiation hazard as well as potential side effects of contrast agents. Thus, they fail to reflect the tumor status timely which may cause potential delays in the assessment of the treatment response and affect the early adjustment of the treatment direction. Blood testing is non-invasive and convenient, which is more suitable as a real-time feedback for treatment efficacy. CA15-3 and CEA are the serum markers that are most commonly used in breast cancer diagnostics but their clinical application is limited due to the low sensitivity and specificity [40]. In contrast, a significant upregulation of RAI14 in TNBC patients was observed in our study and its dynamic changes could precisely reproduce the patients’ therapeutic response at the beginning of chemotherapy, which was in agreement with the imaging assessment results. Taken together, RAI14, as a highly promising serum biomarker, can be used as a complementary indicator for the early diagnosis of TNBC and monitoring the chemotherapeutic efficacy. RAI14 is widely expressed in human tissues and is relevant to the cytoskeletal function. High-throughput sequencing results suggest that a high expression of this gene is associated with the progression of multiple tumors, including BC [29]. Nevertheless, existing studies on serum RAI14 levels are limited and its expression characteristics and corresponding clinical value in TNBC still require further investigation.

The experimental findings suggested that the expression of RAI14 was stable under diverse processing conditions and possessed the potential to become a serum marker. Consistent with the results of the database analysis, serum RAI14 was hyper expressed in TNBC patients with increased levels of RAI14 mRNA. We found that RAI14 expression was markedly linked to the ER, PR, and HER2 status as shown by the results of the TCGA database analysis, a feature that is in agreement with the serum assay results. As we discovered later, serum RAI14 levels were distinctly different in the BC subtypes, in which the highest expression was found in the TNBC subtype along with a great clinical significance. Similar to CA15-3 and CEA levels, there was no elevated serum RAI14 level in all TNBC patients but rather significant individual differences. Through correlation analysis of the clinicopathological factors, it was found that the concentration of RAI14 was positively correlated to tumor volume, CA15-3 and LDH serum levels, which means patients with a larger tumor volume were more likely to have higher RAI14 levels. Other studies [41–44] reported that high-levels of CA15-3 and LDH could affect distant metastasis leading to a poor prognosis of BC patients. Thus, we suggest that the upregulation of RAI14 may contribute to tumor progression. However, no statistical differences were seen in RAI14 levels in BC patients with a variety of clinical stages and lymph node metastasis status in this study; therefore, whether the expression of RAI14 relates to the stage of BC tumor development deserves more attention in our follow-up studies.

RAI14 served as an actin-binding protein, is a major component of the cytoplasmic matrix actin filament network, different from traditional biomarkers [27,45]. CA15-3, expressed in mammary epithelial cells, is a soluble form of the glycoprotein MUC-1. And the high expression of CA15-3 in the cell membrane and cytoplasm, as well as abnormally high serum concentrations, were seen in TNBC [46]. CEA belongs to the family of cell surface glycoproteins that plays an important role in cell adhesion [47] and elevated levels can be observed in a wide range of tumors, including BC. However, there were no apparent differences in the concentrations of CA15-3 and CEA at the beginning of BC [48]. Also, in our experimental results they were found to be of restricted sensitivity and specificity in the diagnosis of TNBC, resulting in the limited application. RAI14 concentrations were much higher in all stages of TNBC patients than in healthy individuals in this study, while the ROC curve results had shown that RAI14 has a good ability to discriminate early TNBC patients and healthy ones, so we considered that RAI14 might function as a proper screening marker for early TNBC more appropriately than C15-3 and CEA. For patients without high CA15-3 levels, RAI14 also showed a high diagnostic efficacy. It is thus clear that RAI14 can be used as a complementary marker to improve the opportunities of screening early TNBCs together with CA15-3.

According to the literature, this is the first research to provide insight into the potential of serum RAI14 levels as a mean to detect chemotherapeutic efficacy. The early efficacy evaluation is of great importance in minimizing the mortality in TNBC, especially to be able to implement timely clinical adjustments of the drug regimen and by this, minimize the risk of tumor progression for patients who are insensitive to therapeutic drugs. A timely and accurate reflection of the genuine disease status is the primary criterion for screening biomarkers. It has been proposed in the literature that the changes of serum CA15-3 and CEA concentrations at the beginning of treatment may be affected by multiple factors and are therefore inappropriate to reproduce the early treatment response correctly [49]; the findings also confirmed the limited use of CA15-3 and CEA in the assessment of patient outcomes at an early phase. Instead of this, the dynamic changes occurring in serum RAI14 levels do reflect the effect of chemotherapy much more precisely than conventional markers. The extended analysis demonstrated that during early chemotherapy, serum RAI14 levels displayed a considerable trend to decrease with an increase in the number of chemotherapy cycles in those who were effective, and the higher the degree of the RAI14 decrease, the better the patient’s response to the treatment; if progression appeared due to drug resistance or lesion metastasis, the serum RAI14 levels did not drop significantly after chemotherapy compared to baseline. The calculation of the RAI14 decline rate provided an excellent and intuitive indicator for clinical efficacy. More so, the results of the conjoint analysis with imaging told us that both RAI14 and tumor volume had very similar changing trends; besides, the decline rate of RAI14 offered an exact judgment of the efficacy and the results were matched with the clinical results, which further confirmed that RAI14 is a more suitable parameter in cancer monitoring than CA15-3 and CEA, as well as providing a more reliable and precise basis for the determination of chemotherapy effects. Furthermore, the basic level of RAI14 is correlated with the outcome of chemotherapy and its detecting efficiency. Patients with high RAI14 baseline levels tended to benefit more from treatment in this study. RAI14 also performed better as a biomarker in therapeutic monitoring in this case and vice versa. The highly malignant and extensive lesions of advanced TNBC are responsible for a more pronounced tumor-killing effect of cytotoxic drugs at the beginning of treatment; simultaneously, since there may be a positive correlation between RAI14 levels and the severity of the disease, patients with high baseline RAI14 levels were inclined to had a more noticeable response to early treatment while RAI14 served a more effective role in chemotherapy monitoring.

In some patients, irregular fluctuations of serum RAI14 levels in conjunction with chemotherapy cycles were observed. To elucidate the causes, we reviewed the medical records and concluded the possible contributors such as chemotherapy resistance, bone marrow suppression, and abnormal liver function. Chemoresistance is one of the main factors responsible for the high mortality rate of TNBC, as drug resistance inhibits the killing effect of chemotherapy on tumor cells [50,51]. The volatility of RAI14 with dosing cycles in some patients fits the changing trend of drug resistance, i.e. the serum concentration of RAI14 increases abnormally after receiving 2 cycles of epi-amycin or paclitaxel and then decreases following a switch in drug regimen. It has been documented [52] that a knockdown of the RAI14 gene can raise the sensitivity of tumor cells to drugs such as epi-amycin and cisplatin, and patients with high RAI14 expression are at higher risk of developing resistance to chemotherapeutic drugs (paclitaxel, epi-amycin, docetaxel, etc.). While the expression of RAI14 was obviously elevated in TNBC, patients were more likely to develop resistance to conventional anti-BC chemotherapeutic drugs such as paclitaxel and epoetin compared to other breast cancer subtypes. As a consequence, the initial therapeutic drugs might not work for the above-mentioned patients, thus, drug resistance occurred which led to a weakened tumor-killing effect of the cytotoxic drugs and therefore, could not effectively inhibit the malignant proliferation of the tumor cells. This led to the invasion and disease progression of TNBC tumors and a parallel increase in their serum RAI14 levels. After replacing the medication plan, the cancer cells were successfully killed, which resulted in a reduction of the tumor lesion and a notable decline in the RAI14 level.

In the efficacy assessment studies, some patients displayed greatly reduced peripheral blood leukocyte and platelet counts after multiple cycles of chemotherapy and that could be clinically judged as being myelosuppression. Chemotherapeutic drugs that are highly lethal to proliferating active cells may cause multilineage myelosuppression by its toxic effects on hematopoietic stem and progenitor cells, leading to impaired bone marrow hematopoiesis. Patients with bone marrow suppression are likely to display not only a reduced number of innate immune cells but also a decreased maturity of the remaining cells, thus preventing them from performing immune surveillance functions properly. Owing to the lack of antigen presentation and other functions, the clearance of tumor antigens by the body’s specific immune system is also inhibited. To sum up, it can be seen that the initial chemotherapy drugs had a beneficial impact on anti-tumor killing and a weak toxic effect in the above patients, so the treatment effect was obvious and the serum RAI14 levels dropped. The toxic side effects of the drugs after multi-stage chemotherapy were gradually obvious, leading to impaired bone marrow hematopoiesis, weakened immune surveillance and clearance of tumor cells by the body’s immune system therefore, cancer progression occurred and the patient’s RAI14 serum concentration subsequently increased.

In some other cases, AST and ALT serum concentrations were abnormally increased through chemotherapy, suggesting damaged liver function. The impairment of cellular structure and function of the liver, which is the main organ of biotransformation, is one of the most common toxic side effects of chemotherapy due to the untargeted cytotoxic effect of the drugs. When attacked by cytotoxic substances, hepatocytes are prevented from growth, differentiation, and nutrient supply, etc [53]. Moreover, the drug can lead to hepatic necrosis *via* ADCC, an antigen or semi-antigen that activates the body’s specific immune system. Hence, the liver biotransformation function was diminished which resulted in reduced catabolism of RAI14 protein and ultimately its concentration appeared to be increased. Following several hepatoprotective treatments, the patients’ hepatic function was restored and soon the RAI14 levels were dropped. Taken together, the application of traditional tumor markers in chemotherapy monitoring is relatively limited whereas the changes of the RAI14 levels reflect not only drug efficacy and organ damage but furthermore also bone marrow suppression, etc. These data are much more informative for clinical patient evaluation with regard to an adjustment of drug dosage and formulation of the medication plan.

## 5. Conclusion

In summary, the determination of serum RAI14 levels has a complementary effect to determine CA15-3 levels and the combined detection of both parameters greatly enhanced the identification rate of patients with early TNBC, while tracking the RAI14 level trends during chemotherapy can be applied for the early assessment of treatment efficacy and thus, opens up the possibility of a continuation of an effective treatment or discontinuation of ineffective regimens caused by erroneous judgments of efficacy. This research highlighted the potential of RAI14 in TNBC chemotherapy monitoring. Dynamic changes of RAI14 levels represent a precise parameter for the early treatment of TNBC and provide a more direct and reliable reference for efficacy assessment in comparison to the determination of CA15-3 levels and tumor diameter. There was an additional link between baseline levels and the response to treatment. Patients with higher levels tended to have greater decreases in RAI14 levels after dosing, which predicted that there would be a larger benefit after treatment. Taken together, RAI14 is a key player in the examination of the treatment effects as well as a basis for clinical modification of drug regimens.

## Supplementary Material

Supplemental Material

## Data Availability

The datasets during the current study are available from the corresponding author on reasonable request.
