# Supplementary material for: The dual-crosslinked prospective values of RAI14 for the diagnosis and chemosurveillance in triple negative breast cancer
Source: Ann Med. 2023 Mar 7;55(1):820–36. doi: 10.1080/07853890.2023.2177722 (PMC10795645; doi:10.1080/07853890.2023.2177722)
Supplement: Supplemental Material [file IANN_A_2177722_SM5438.doc]

**The Dual-crosslinked Prospective Values of RAI14 for the Diagnosis and Chemosurveillance in Triple Negative Breast Cancer**

**A table of contents**

| Contents  Supplementary Materials ＆ Methods  Supplementary Tables  Supplementary Figures |
| --- |

**Supplementary Methods ＆ Materials**

***Immuoprecipitation***

The Co-IP Kit (Thermo Fisher Scientific, Waltham, MA, United States) was designed for the detection of interactions among components of the target protein complex. In total, all the following steps were performed according to the kit instructions. The antigen–antibody complexes were finally eluted. A portion of the protein solution is sent for mass spectrometry (UW BGI) to detect the interacting protein with CPN1. A portion was subjected to SDS-PAGE.

***ELISA assay***

Serum RAI14 concentrations of patients and controls were determined by enzyme-linked immunosorbent assay.Selected commercial kits (Shanghai Jianglai Biotechnology Co. Ltd, China).The laboratory procedure was operated according to strict instructions. Dilution of standards in multiples of the instructions to 1000pg/mL,500pg/mL ,250pg/mL,125pg/mL,62.5pg/mL,31.2pg/mL,15.6pg/mL. The standard dilution (0pg/mL) was used as blank wells. After adding serum sample and standard solution to a 96-well microplate coated with capture antibody and incubating at 37℃, discard the liquid, add working solution A, B and TMB substrate solution in turn, wash the plate with an automatic plate washer (TECAN, HydroFlex) before each addtion of reagents, add the reagant and incubate, then add the termination solution and incubate. The sample Optical Density of the solutions was read at 450nm wavelength with an enzyme marker (Thermo Mutiskan FC) to caculate the concentration of RAI14 by the standard curve of ELISA.

***Electrochemiluminescence***

The concentration of CA15-3 and CEA in serum was measured through a Roche electrochemiluminescence automated immunoassay system (Roche Cobas 801). CA15-3, CEA and related buffer reagents were provided by Roche and operated in strict accordance with the specifications of the manufacturer’s instructions.The normal ranges of CA15-3, CEA and CA125 in serum are 0-25U/mL, 0-5µg/L and 0-35U/L, respectively.

***Colorimetry***

We selected a colorimetric method to test the serum LDH concentration by a Roche Cobas c701 analyzer equipped with special reagents.The normal serum LDH concentration ranges from 120 to 250 U/L.

***Blood sample analysis***

The numbers of white blood cells and platelets were determined using a hemocytometer (Sysmex，XN-9000 Plus_L1). The normal value of neutrophils is (1.8-6.3)×10^9/L, and the normal lymphocyte range is (1.5-3.3)×10^9/L.

***Statistical analysis***

The experimental data of this study was processed by statistical softwares SPSS 25.0, GraphPad Prism 9.0 for analysis. The clinicopathological information of the patients was statistically described in the form of categorical variables and analyzed by chi-square test. The continuous quantitative data obeying normal distribution was presented as mean ± standard deviation. For the comparison between groups, independent sample t-test was used, and Pearson correlation analysis was selected for the two variables; The median represented non-normally distributed quantitative data, which used the Mann-Whitney U test for inter-group comparison, and Spearman correlation analysis was picked. Alternatively, we analyzed the diagnostic efficacy of markers and their rate of concentration decline in the assessment of efficacy using ROC curves. P<0.05 meant statistically significant.

| **Table S1.**Clinical information for patients in prevalence assessment study | | | | | | | | | | | | | | | | | | | | | | |
| --- | --- | --- | --- | --- | --- | --- | --- | --- | --- | --- | --- | --- | --- | --- | --- | --- | --- | --- | --- | --- | --- | --- |
| Parameters | |  | | | | | | | | |  |  | N | | | | Age（M） | | | | | |
| Clinical Diagnosis | | | |  | | | | | | |  |  |  | | | |  | | | | | |
|  | | Breast cancer | | | | | | | | |  |  | 116 | | | | 51 | | | | | |
|  | | Benign breast disease | | | | | | | | |  |  | 30 | | | | 42 | | | | | |
|  | | Healthy | | | | | | | | |  |  | 30 | | | | 39 | | | | | |
| Tumor size（cm） | |  | | | | | | | | |  |  |  | | | |  | | | | | |
|  | | ≤2 | | | | | | | | |  |  | 43 | | | | 50 | | | | | |
|  | | 2-5 | | | | | | | | |  |  | 67 | | | | 51 | | | | | |
|  | | ≥5 | | | | | | | | |  |  | 6 | | | | 57.5 | | | | | |
| Lymph node metastasis | | | | | | |  | | | |  |  |  | | | |  | | | | | |
|  | | Absent | | | | | | | | |  |  | 46 | | | | 51 | | | | | |
|  | | Present | | | | | | | | |  |  | 70 | | | | 51 | | | | | |
| TNM Stages | |  | | | | | | | | |  |  |  | | | |  | | | | | |
|  | | Ⅰ-ⅢA | | | | | | | | |  |  | 104 | | | | 51 | | | | | |
|  | | ⅢB-Ⅳ | | | | | | | | |  |  | 12 | | | | 60.5 | | | | | |
| Molecular subtypes | | | | |  | | | | | |  |  |  | | | |  | | | | | |
|  | | Luminal A | | | | | | | | |  |  | 20 | | | | 51.5 | | | | | |
|  | | Luminal B | | | | | | | | |  |  | 20 | | | | 49 | | | | | |
|  | | HER2+ | | | | | | | | |  |  | 30 | | | | 54 | | | | | |
|  | | TNBC | | | | | | | | |  |  | 46 | | | | 50.5 | | | | | |
| ER | |  | | | | | | | | |  |  |  | | | |  | | | | | |
|  | | positive | | | | | | | | |  |  | 40 | | | | 50 | | | | | |
|  | | negative | | | | | | | | |  |  | 76 | | | | 52.5 | | | | | |
| PR | |  | | | | | | | | |  |  |  | | | |  | | | | | |
|  | | positive | | | | | | | | |  |  | 34 | | | | 49.5 | | | | | |
|  | | negative | | | | | | | | |  |  | 82 | | | | 51.5 | | | | | |
| HER2 | |  | | | | | | | | |  |  |  | | | |  | | | | | |
|  | | positive | | | | | | | | |  |  | 50 | | | | 51 | | | | | |
|  | | negative | | | | | | | | |  |  | 66 | | | | 51 | | | | | |
| **Table S2.**Clinical data of patients in efficacy monitoring study | | | | | | | | | | | | | | | | | | | | | | |
|  | | | | | | | | | | | | | | | | | | | | | | |
| Parameters |  | | | | | | | |  | | | |  | | N | | | | Age（M） | | | |
| Tumor size（cm） | | |  | | | | | |  | | | |  | | | |  | |  | | | |
|  | ≤2 | | | | | | | |  | | | |  | 14 | | | | | | 56 | | |
|  | 2-5 | | | | | | | |  | | | |  | 33 | | | | | | 53 | | |
|  | ≥5 | | | | | | | |  | | | |  | 10 | | | | | | 50 | | |
| Lymph node metastasis | | | | | | |  | |  | | | |  | | | |  | |  | | | |
|  | Absent | | | | | | | |  | | | |  | | 48 | | | | | | | 54 |
|  | Present | | | | | | | |  | | | |  | | | 9 | | | | | | 52 |
| Distant metastasis | | | | | |  | | |  | | | |  | | | |  | |  | | | |
|  | Presant | | | | | | | |  | | | |  | | 28 | | | | | | | 54 |
|  |  | | | | | | | | Bone metastases | | | |  | 6 | | | | | | | | 54.5 |
|  |  | | | | | | | | Lung metastases | | | |  | 10 | | | | | | | | 56.5 |
|  |  | | | | | | | | Liver metastases | | | |  | 5 | | | | | | | | 61 |
|  |  | | | | | | | | Pleural metastases | | | |  | 7 | | | | | | | | 48 |
|  | Absent | | | | | | | |  | | | |  | 29 | | | | | | | | 54 |
| TNM stages |  | | | | | | | |  |  | | | |  | | | | |  | | | |
|  | Ⅰ-ⅢA | | | | | | | |  | | | |  | 24 | | | | | | | 54.5 | |
|  | ⅢB-Ⅳ | | | | | | | |  | | | |  | 33 | | | | 57 | | | | |
| Evaluation of efficacy | | | | | | | |  |  | | | |  |  | | | | | | |  | |
|  | PR+CR+SD | | | | | | | |  | | | |  | 46 | | | | | | | 52.5 | |
|  | PD | | | | | | | |  | | | |  | 11 | | | | | | | 56 | |

| **Table S3**.Performances of biomarkers for the diagnosis of TNBC | | | | | | | | | | | | | |  | |
| --- | --- | --- | --- | --- | --- | --- | --- | --- | --- | --- | --- | --- | --- | --- | --- |
|  | AUC | | | | Sensitivity (%) | | |  | | | Specificity (%) | | | | |
| RAI14 | 0.934 | | | | | 86.7 |  | 90.0 | | | | |  | | |
| CA15-3 | 0.836 | | | | | 67.4 |  | 93.3 | | | | |  | | |
| CEA | 0.636 | | | | | 80.4 |  | 46.7 | | | | |  | | |
| CA125 | 0.566 | | | | | 76.1 |  | 40.0 | | | | |  | | |
| RAI14+CA15-3 | 0.948 | | | | | 87.0 |  | | | | 93.3 | |  | | |
| RAI14+CEA | 0.948 | | | | | 84.8 |  | | | | 93.3 | |  | | |
| RAI14+CA125 | 0.936 | | | | | 87.0 |  | | | | 90.0 | |  | | |
| RAI14(Early-stage) | | 0.929 | | | 85.7 | |  | | | | | 90.0 | | |  |
| RAI14(CA15-3<25U/mL) | | | 0.908 | 81.8 | | | | |  | 90.0 | | | | | |


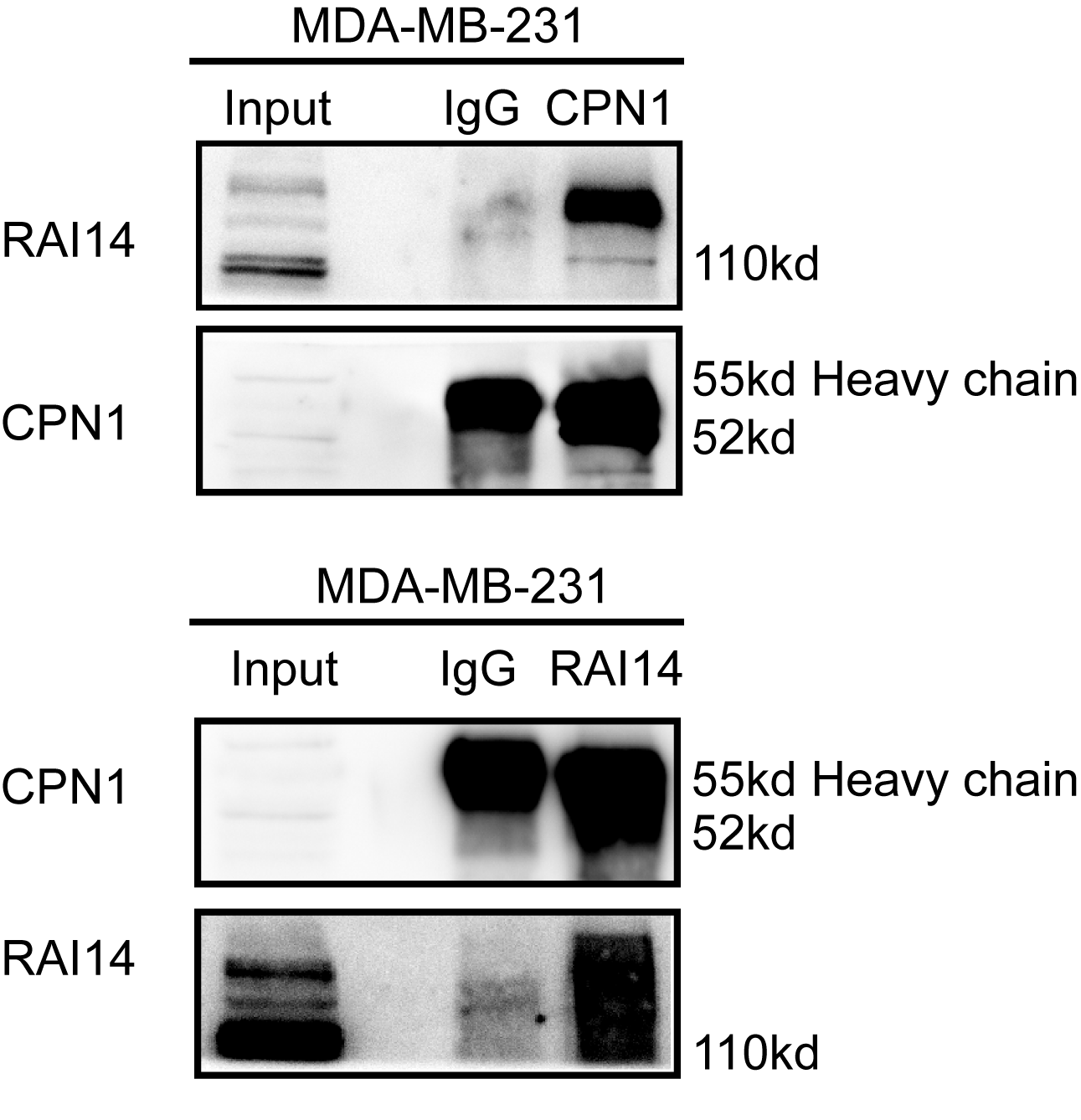


**Fig. S1** Validation of RAI14 interacting with CPN1.(CO-IP)


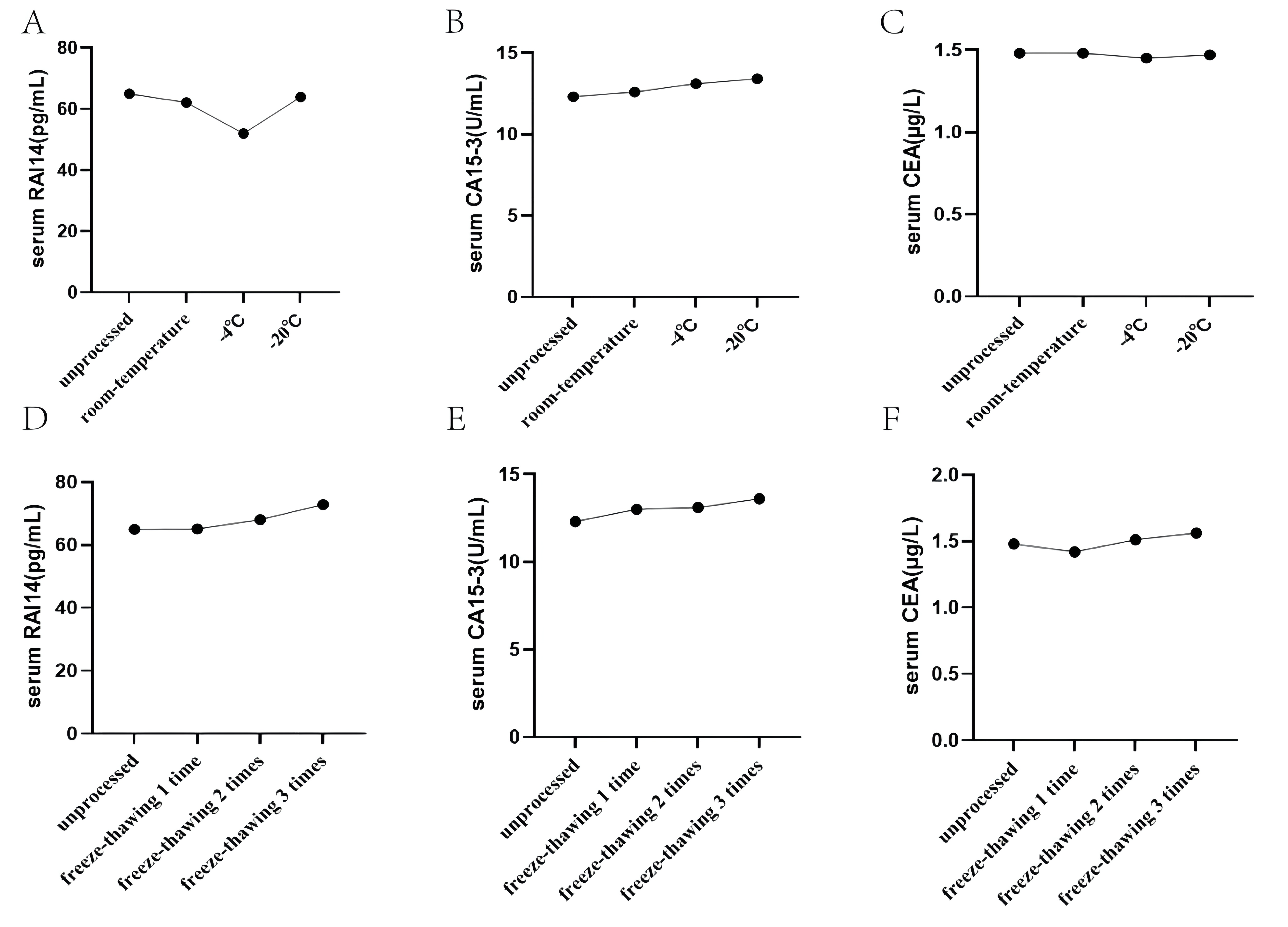


**Fig. S2** Serum stability of RAI14.A-C:Changes in serum concentrations of RAI14(A),CA15-3(B) and CEA(C) after 24h processing at different temperatures;D-F:Variation of serum RAI14(D), CA15-3(E) and CEA (F)concentrations after repeated freeze-thaw treatments.


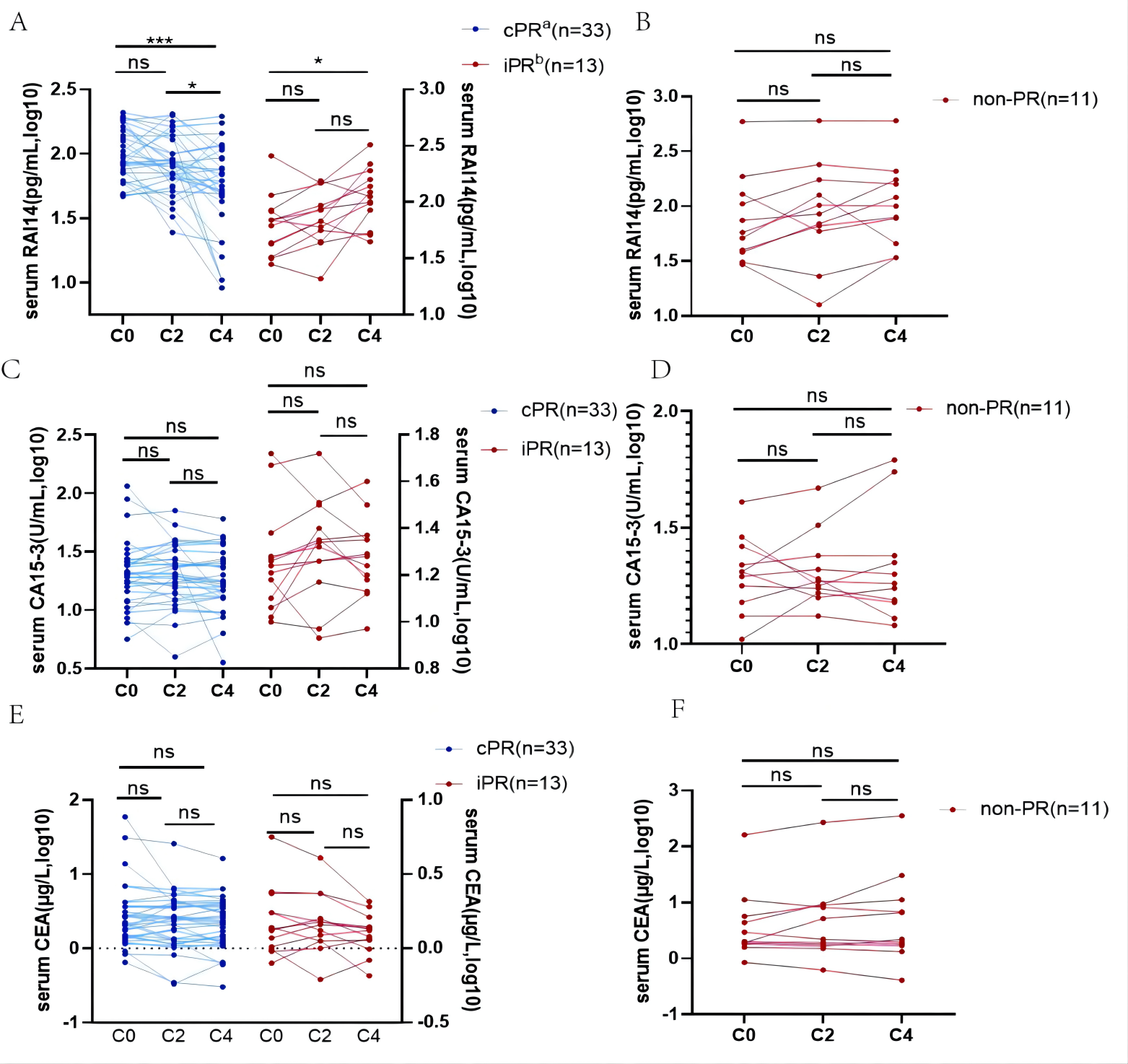


**Fig. S3** Patterns of changes in serum RAI14, CA15-3 and CEA levels in patients during chemotherapy. A, C, E: Patterns of changes in the expression levels of serum RAI14(A), CA15-3(C) and CEA(E) in PR group patients during chemotherapy cycles; B, D, F: Trends of serum RAI14(B), CA15-3(D) and CEA (F)concentrations before and after chemotherapy in the non-PR group. a: cPR=consistent with PR; b: iPR= inconsistent with PR.


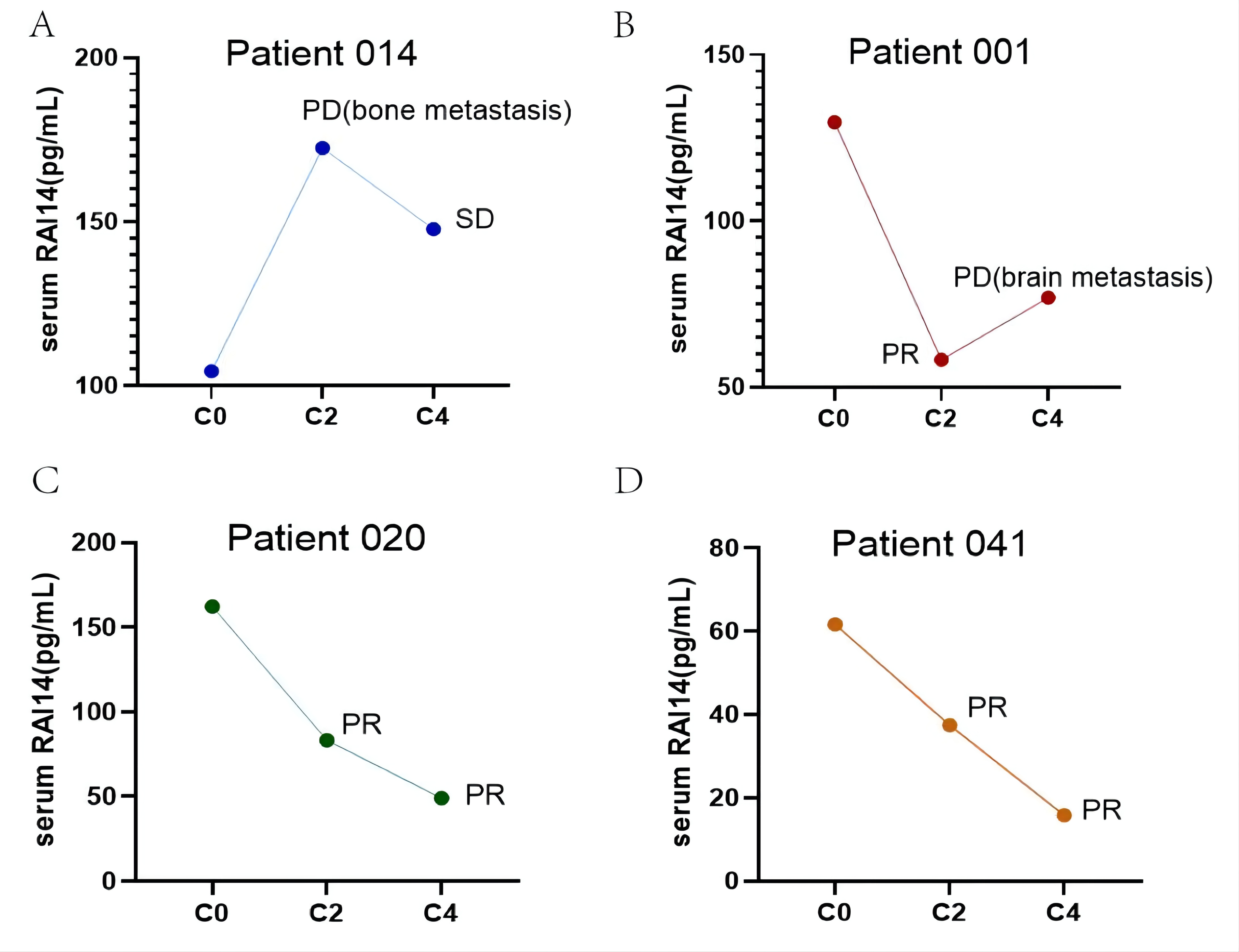


**Fig. S4** Consistency analysis of differences in RAI14 levels with clinical efficacy assessment.A-D:The evaluation of clinical efficacy and the changes of serum RAI14 levels after chemotherapy in C0, C2 and C4 stages among four patients.


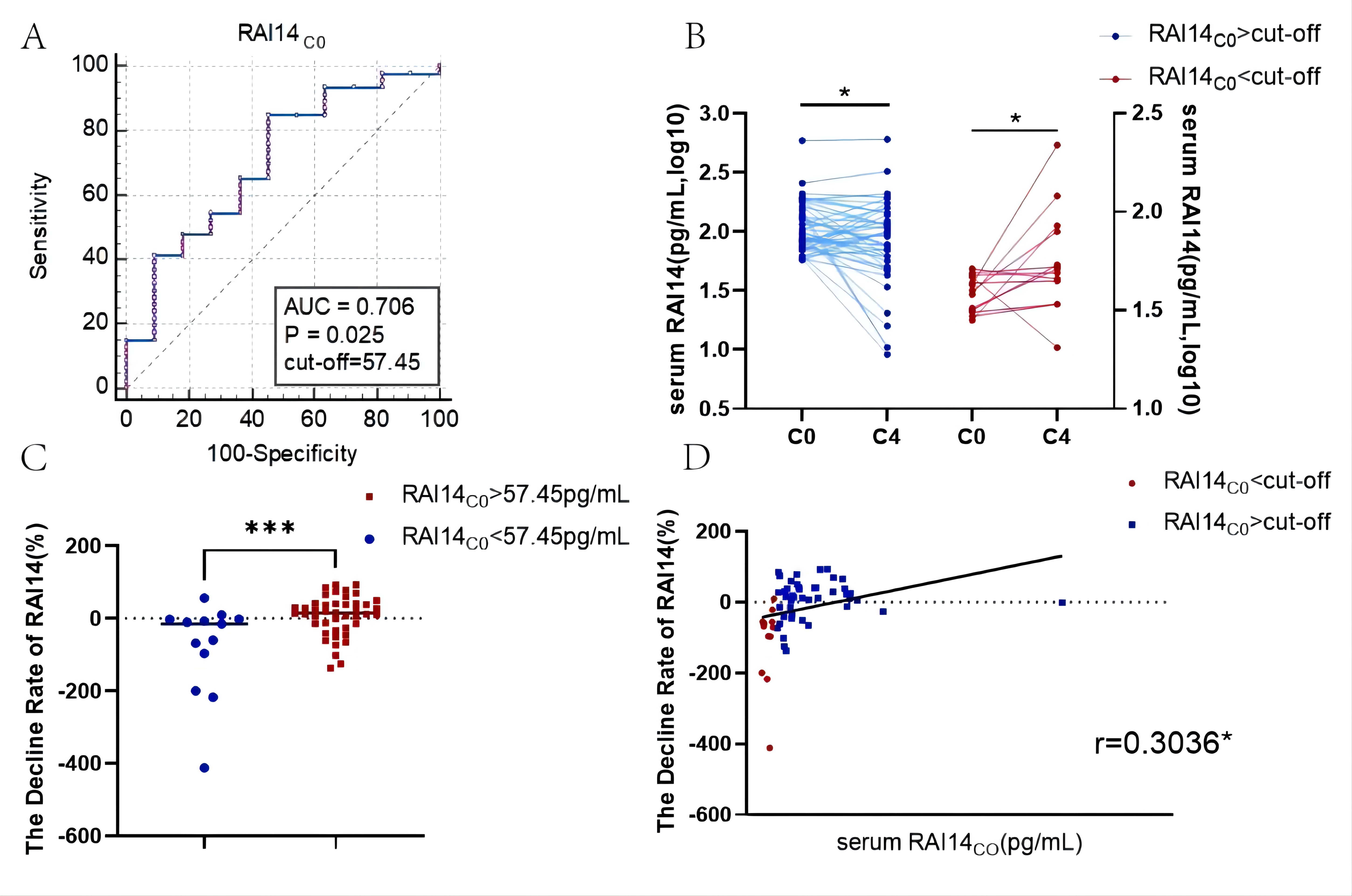


**Fig. S5** The relationship between patients' initial RAI14 levels and their efficacy.A:ROC curves of RAI14 baseline levels of patients in PR, non-PR groups;B:Patterns of changes in serum RAI14 concentrations after chemotherapy in patients with different baseline levels;C:Distribution of decline rates after chemotherapy of patients with different RAI14_C0_ levels;D:Correlation analysis of RAI14 baseline and the rate of decline.


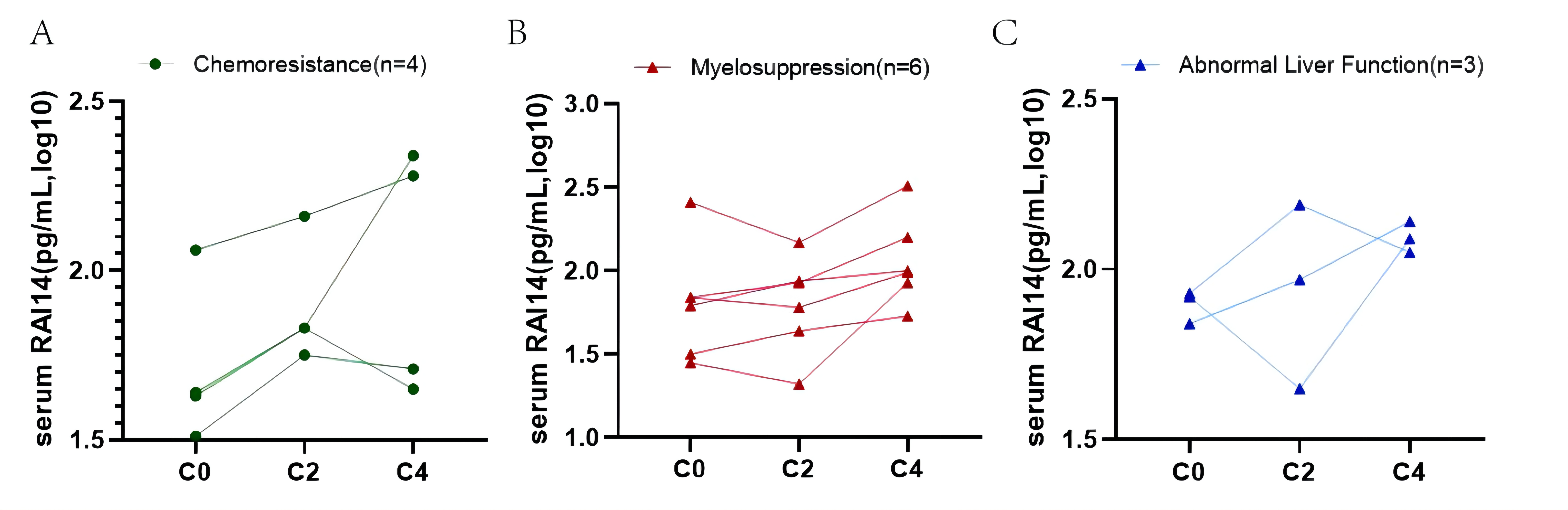


**Fig. S6** Analysis of irregular variations of RAI14 concentration.A-C:The pattern of change of serum RAI14 concentration with drug administration cycle in chemotherapy-resistant group, myelosuppression group and abnormal liver function group.
